# Comorbidities and Outcomes among Females with Non-Alcoholic Fatty Liver Disease Compared to Males

**DOI:** 10.3390/biomedicines10112908

**Published:** 2022-11-12

**Authors:** Naim Abu-Freha, Bracha Cohen, Sarah Weissmann, Reut Hizkiya, Reem Abu-Hammad, Gadeer Taha, Michal Gordon

**Affiliations:** 1 The Institute of Gastroenterology and Hepatology, Soroka University Medical Center, Beer-Sheva 84101, Israel; 2 The Faculty of Health Sciences, Ben-Gurion University of the Negev, Beer-Sheva 84101, Israel; 3 Soroka Clinical Research Center, Soroka University Medical Center, Beer-Sheva 84101, Israel; 4 Internal Medicine Division, Soroka University Medical Center, Beer-Sheva 84101, Israel; 5 Department of Gastroenterology, Rambam Health Care Campus, Haifa 31096, Israel

**Keywords:** fatty liver, cirrhosis, females, gender, liver

## Abstract

Sex-based medicine is an important emerging discipline within medicine. We investigated the clinical characteristics, complications, and outcomes of Nonalcoholic Fatty Liver Disease (NAFLD) in females compared to males. Demographics, comorbidities, malignancy, complications, outcomes, and all-cause mortality of NAFLD patients older than 18 years were analyzed. The data were extracted using the MDClone platform from “Clalit” in Israel. A total of 111,993 (52.8%) of the study subjects were females with an average age of 44.4 ± 14.7 years compared to 39.62 ± 14.9 years in males, *p* < 0.001. Significantly higher rates of hypertension, diabetes mellitus, obesity, dementia, and thyroid cancer and lower rates of ischemic heart disease (22.3% vs. 27.3%, *p* < 0.001) were found among females. Females had a higher rate of cirrhosis, 2.3% vs. 1.9%, *p* < 0.001, and a lower rate of hepatocellular carcinoma, 0.4% vs. 0.5%, *p* < 0.001. In the multivariate analysis, a relationship between age, diabetes mellitus, and cirrhosis development were found among males and females. A lower age-adjusted mortality rate was found among females, 94.5/1000 vs. 116/1000 among males. In conclusion, older age at diagnosis, higher rates of hypertension, diabetes mellitus, obesity, cirrhosis, and a lower age-adjusted all-cause mortality rate were found among females with NAFLD.

## 1. Introduction

Non-Alcoholic Fatty Liver Disease (NAFLD) is the most common liver disease, affecting around 25–30% of the population in some countries, with the highest prevalence in the Middle East and South America and the lowest in Africa [1,2,3]. The prevalence is increased among older people as well as those diagnosed with diabetes or obesity, possibly even reaching 60% of these populations [1]. As a common chronic liver disease, NAFLD frequently causes cirrhosis [4]. Moreover, a large part of chronic liver disease complications such as hepatocellular carcinoma (HCC), liver transplantation, and mortality result from NAFLD [5].

Sex-based medicine is a relatively new and important field of research that has emerged in the last decade. The impact of sex on illnesses can manifest as differences in prevalence, disease course, and outcomes. In the gastroenterology and hepatology field, significant sex-based differences have been found in colorectal cancer development and incidence, anatomical site, survival, indications and upper endoscopy findings [6,7,8]. Sex-related differences in epidemiology, disease progression, and treatment strategies of liver diseases have also been reported [9]. Drug toxicity and drug-dose gender gaps have been widely reported between males and females. Women have higher rates of autoimmune hepatitis (70–90% of cases are women), primary biliary cholangitis, and hepatocellular carcinoma. Women, however, have lower rates of primary sclerosing cholangitis, with a male:female ratio of 7:3 [9]. Sex differences have also been found regarding alcohol consumption and alcohol-associated liver disease: the prevalence of severe alcohol use disorder was reported in 18.3% of men and 9.7% of women in the USA, with women developing more severe alcohol-associated liver disease at lower levels of exposure compared to their male counterparts [10].

Hepatocellular carcinoma (HCC) is one of the feared complications of chronic liver diseases, and significant sex-related differences have been previously reported. HCC is a liver neoplasm with a multifaceted nature of causes, risk factors and genetic alterations [11,12]. Females present with HCC at an older age and with a higher number of HCC and hypertension cases in their family histories than males [13,14]. In addition, females with HCC were more likely to undergo HCC surveillance, have smaller tumor sizes at diagnosis, and have less vascular involvement [13,14].

Only scant data were published regarding sex-related disparities of NAFLD patients, and it is a relatively under-researched field [15]. On average, females make up a higher percentage of NAFLD cases than males [15]. Sex-related differences have been found in adolescents, with a higher prevalence of NAFLD (16.3% vs. 10.1%) and central obesity (33.2% vs. 9.9%) reported among females [16]. In general, the prevalence of NAFLD is higher among men and postmenopausal women than among women of reproductive age, possibly suggesting a hormonal protective role [17].

This study aimed to investigate and determine the disparities in comorbidities (particularly metabolic syndrome), laboratory data, liver-related outcomes, and mortality of female patients with NAFLD compared to males with NAFLD. Understanding these disparities is crucial for the diagnosis, follow-up, treatment, and surveillance of patients with NAFLD.

## 2. Materials and Methods

### 2.1. The Materials Study Design and Patients

This was a retrospective study that included patients aged 18 years or older diagnosed with NAFLD between the years 2000 and 2021. NAFLD patients were identified by having an ICD 10 code of K76.0 at any time in their chronic disease list (according to community data or hospital data). A total of 9353 patients with liver-related comorbidities including alcoholic liver disease, hepatitis B, and hepatitis C were excluded from our population. The sample of NAFLD patients was then subdivided according to sex.

### 2.2. Data Collection

The data was extracted from Clalit Health Services (CHS) using Clalit’s data-sharing platform powered by MDClone (https://www.mdclone.com (accessed on 7 October 2022)). CHS is the largest health maintenance organization in Israel, with about 4.7 million insured residents.

Demographics, laboratory data, complications, outcomes, and mortality data were retrospectively collected from NAFLD patients. Laboratory data included complete blood count, alanine transaminase (ALT), aspartate transaminase (AST), bilirubin, albumin, and international normalized ratio (INR), taken from blood samples at or nearest to the time of diagnosis. In addition, the Fib-4 and APRI scores were calculated at the time of diagnosis. Comorbidities including metabolic syndrome, cancer, and other common diseases were collected from computerized files according to the specific ICD-10 codes. Outcomes including cirrhosis, hepatocellular carcinoma, liver transplantation, and all-cause mortality were collected according to the ICD-10 codes as well. All collected data were compared between males and females.

### 2.3. Statistical Analysis

Data are presented as mean ± standard deviation (SD) for continuous variables and as a percentage (%) of the total for categorical variables. Univariate analyses were performed using independent T-tests for continuous variables and chi-square tests for categorical variables. We used logistic regression models to examine the multivariate relationships between risk factors and the odds of death. Before introducing the variables into the model, multicollinearity of the variables was examined using the Variance Inflation Factor (VIF) statistic. The variables found to be significant in the univariate analysis were introduced into the multivariate model one after the other, and included age at diagnosis, gender, diabetes mellitus, cirrhosis, hepatocellular carcinoma, and esophageal varices. We calculated the all-cause mortality death rate among the groups, subdivided by age. The all-cause mortality death rate was age-adjusted using a general population control group from Clalit (452,012 people). All statistical analyses were performed using IBM SPSS version 26 (Chicago, IL, USA). *p*-values less than 0.05 were considered statistically significant. The study was carried out in accordance with the principles of the Helsinki Declaration. The study protocol was approved by the Institutional Helsinki Committee, approval number 198-21-SOR.

## 3. Results

### 3.1. Patients

The baseline characteristics, comorbidities, and malignancy rates among NAFLD patients are presented in Table 1. A higher percentage of our cohort was female (n = 111,993, 52.8%). Females were diagnosed at an older age, 44.4 ± 14.7 years, compared to males, 39.62 ± 14.9 years, *p* < 0.001. Higher rates of hypertension, diabetes mellitus, obesity, and dementia were observed among female NAFLD patients, 60.7% vs. 53.5%, 24.7% vs. 21.6%, 64.2% vs. 52.7%, 5.2% vs. 2.9%, respectively, *p* < 0.001. However, lower rates of ischemic heart disease and chronic renal failure were found among females, 22.3% vs. 27.3%, 11.2% vs. 14.8%, respectively, *p* < 0.001. A higher rate of thyroid carcinoma and a lower rate of kidney carcinoma were observed among females, 1% vs. 0.4%, *p* < 0.001, and 0.6% vs. 1% *p* < 0.001. No significant difference regarding other malignancies was found between the two populations. We found that NAFLD was diagnosed before most other metabolic syndrome-related diseases. A total of 99.5% of patients were diagnosed with diabetes mellitus after being diagnosed with NAFLD (0.5% of males and 0.5% of females were diagnosed with diabetes before NAFLD (*p* = 0.966). Only 8% of males and 8.5% of females were diagnosed with obesity before NAFLD, compared to 92% of males and 91.5% of females who were diagnosed after being diagnosed with NAFLD, *p* = 0.001. Hypertension, dyslipidemia, CIHD, and CVA were also diagnosed more commonly among males after NAFLD diagnosis compared to females.

### 3.2. Laboratory Results among the Study Groups

The laboratory results are summarized in Table 2. Significant differences between females and males were found regarding several lab values: AST (30.7 ± 36 vs. 33.8 ± 39, *p* < 0.001), ALT (34.2 ± 38.6 vs. 47.14 ± 52.9, *p* < 0.001) GGT (51.89 ± 84 vs. 61.45 ± 100, *p* < 0.001) and albumin (4.22 ± 1.4 vs. 4.42 ± 1.76, *p* < 0.001). In addition, lower values of APRI (0.36 ± 0.66 vs. 0.44 ± 0.83, *p* < 0.001) but higher FIB-4 levels were found among females (1 ± 1 vs. 0.96 ± 1.1, *p* < 0.001). All statistical analyses were performed using IBM SPSS version 26 (Chicago, USA). *p*-values less than 0.05 were considered statistically significant.

### 3.3. Liver-Related Outcomes and All Cause-Mortality

The liver-related outcomes and all-cause mortality rates are summarized in Table 3. More females were diagnosed with cirrhosis (2.3% vs. 1.9%, *p* < 0.001), but at an older age compared to males (65.9 ± 12.3 years vs. 63.4 ± 13.7 years, *p* < 0.001, respectively). Lower rates of HCC and liver transplantation were found among females (0.4% vs. 0.5%, *p* < 0.001, 0.07% vs. 0.11%, *p* < 0.003, respectively). No statistical difference was found regarding esophageal varices, esophageal variceal bleeding, spontaneous bacterial peritonitis, and hepatorenal syndrome between males and females. There was a significantly higher rate of all-cause mortality among females compared to males (11.4% vs. 10.2%, *p* < 0.001). The age-adjusted mortality rate was calculated in our cohort using a reference control group of non-NAFLD patients. The all-cause age-adjusted mortality rate was lower among females compared to males (94.5 patients per 1000 female NAFLD patients compared to 116 patients per 1000 male NAFLD patients). The cirrhosis and all-cause mortality rates according to age group are presented in Table 4 and Table 5. A lower rate of liver transplantation was performed in females compared to males (0.07% vs. 0.11%, *p* = 0.003).

### 3.4. Factors Associated with Cirrhosis and All-Cause Mortality

The multivariate analysis regarding cirrhosis development among males and females is presented in Table 6. A relationship between age, diabetes mellitus, and cirrhosis was found among males and females with NAFLD. A significant relationship between obesity and cirrhosis was found among males but not females.

A multivariate model for the risk of death among NAFLD patients included in our study is shown in Table 7. Age at diagnosis, gender, diabetes mellitus, cirrhosis hepatocellular carcinoma, and esophageal varices were found to be risk factors for death among NAFLD patients in the univariate and multivariate analyses, with odds ratios of 1.125, 1.382, 2.648, 4.016, 9.086 and 2.021, *p* < 0.001, respectively.

## 4. Discussion

This study included more than 200,000 NAFLD patients (52.8% female). We found (1) females were diagnosed with NAFLD at an older mean age than males, (2) females had higher rates of comorbidities including metabolic syndrome, hypertension, diabetes mellitus, and obesity than their male counterparts, (3) females had a higher rate of thyroid carcinoma but no significant difference in rates of other cancers, (4) female patients had higher rates of cirrhosis than males and had higher all-cause mortality rates than males, (5) age and diabetes were found to be predictors for cirrhosis among males and females, but obesity was found to be a predictor for cirrhosis only among males, not females, and finally, (6) diabetes mellitus, cirrhosis, and HCC were found to be predictors of death among female NAFLD patients.

Sex-related differences in the context of NAFLD could be attributed to several factors: differences in body structure, behavioral risk factors, comorbidities, metabolic factors, genetics, and hormonal effects.

The body structures of females and males are inherently different. Differences in fat storage, fat metabolism, and health risks of obesity among females and males have been noted [18]. All of these differences could influence the prevalence of NAFLD among females and may have an impact on the clinical course and complications of the disease.

Behavioral risk factors such as smoking, alcohol and food consuming habits could also have an impact on the development of NAFLD. These differences in habits could be co-factors for NAFLD development and progression. Smoking, alcohol use, and fast food consumption are more common among males compared to females [19,20,21,22,23,24]. Despite these differences, the prevalence of NAFLD, cirrhosis development, and all-cause mortality are more common among females, possibly indicating other factors are more dominant influencers of NAFLD among females.

NAFLD is considered as the hepatic manifestation of metabolic syndrome and has a strong relationship with obesity. The chronological relationship between NAFLD and comorbidities is still unclear. In particular, the impact each has on the other, and the causal relationship between the two are still unknown. In our study, the rates of diabetes mellitus, hypertension, and obesity were higher among females than males. Most likely, diabetes mellitus and obesity influence the rate of disease progression of cirrhosis and all-cause mortality rates. In our study, diabetes mellitus was found to be a predictor for cirrhosis among both males and females, while obesity was found to be a predictor for cirrhosis among males only.

Several animal studies have demonstrated sexually dimorphic hepatic genes associated with NAFLD. These genes, related to lipid metabolism, drug metabolism, and glucose homeostasis, impact the severity of cirrhosis and inflammation and are risk factors for the onset, progression, and treatment response of NAFLD [17].

Another critical factor that could contribute to sex differences in NAFLD is the hormonal differences between males and females. Estrogen is a vital sex hormone that not only regulates the female reproductive system but also contributes to several biological functions and protection from different diseases.

In a rodent model, the peak serum tumor necrosis factor-alpha (TNF-a), a proinflammatory cytokine, in the liver was twice as high in rodents who received estrogen compared to controls. This study concluded that estrogen sensitizes Kupffer cells to lipopolysaccharide (LPS), resulting in increased toxic mediator production [25]. This pro-inflammatory and toxic mediator production could also affect the progression of liver diseases such as NAFLD. Hormonal, inflammatory, and oxidative stress factors are part of a complex cascade of NAFLD pathogenesis with sex-related differences [17].

Our results show females are diagnosed with NAFLD about five years later than their male counterparts. This could be explained by the protective estrogen effect from NAFLD, which is lost in postmenopausal women. This is consistent with increasing NAFLD rates with age in women [26,27]. Our findings supported this theory: 34.7% of our female patients were diagnosed with NAFLD at age fifty or older, compared to 25.7% of males.

With regard to comorbidities, we found higher rates of diabetes mellitus, hypertension, and obesity among female NAFLD patients but a lower rate of ischemic heart disease. This finding could be related to the protective effect of estrogen on cardiovascular disease incidence among women [27].

Our study found a higher rate of thyroid malignancy and a lower rate of HCC among females compared to males. Previous studies showed disparities in HCC among females compared to males in terms of undergoing HCC surveillance, tumor size at diagnosis, and vascular involvement [13,14]. Previous studies showed that older age, male sex, the severity of compensated cirrhosis at presentation, and sustained activity of liver disease are important predictors of HCC [28,29,30].

Our study demonstrated that higher rates of cirrhosis development in females, despite an older age at diagnosis and shorter exposure to the steatosis process in females. Hormonal effects and comorbidities such as diabetes and obesity may influence the progression of fibrosis. Whether or not sex is a risk factor for the progression of fibrosis is a controversial issue with conflicting findings across differently designed studies [27]. However, adjusting the cirrhosis rate according to the different age groups, we found a slightly lower rate of cirrhosis among most of the female age groups.

The all-cause age-adjusted mortality rate was lower among females in our study. In addition, a lower rate of HCC was found among females, though there was no significant difference in other complications such as esophageal varices and hepatorenal syndrome. Lower rates of HCC in females may account for the decreased rate of the all-cause mortality.

One of this study’s limitations is the lack of availability of data on liver-specific causes of mortality. This makes it difficult to understand the difference in mortality rate, as it is possibly related to other comorbidities. Nevertheless, in the multivariate analysis, the factors with a significant impact on death were age at diagnosis, gender, diabetes mellitus, cirrhosis, and HCC.

To summarize, significant differences were found between females and males in terms of comorbidities, liver-related outcomes, and all-cause mortality rates. Understanding these differences in depth is crucial for prevention, early diagnosis, interventions, and treatment of NAFLD. Special consideration may be required for females in order to decrease the rate of cirrhosis and all-cause mortality. Additional studies are needed before specific interventions can be carried out; however, the practical implication of the present study lie in increasing awareness about the disparities between NAFLD development and outcomes in males and females.

This study is further strengthened by the use of national-based cohort data with a large number of included patients. However, some limitations should be mentioned. The retrospective design of the study design based on an electronic health file database prevented our ability to differentiate between NAFLD and NASH and there was no data regarding liver biopsy or fibrosis grade available.

## 5. Conclusions

In conclusion, significant differences were found between males and females with NAFLD regarding the age of diagnosis, comorbidities, liver-related complications and all-cause mortality.

## Figures and Tables

**Table 1:** Baseline characteristics, comorbidities, and malignancy among the study groups.

	Males with NAFLD 99,962 (%)	Females with NAFLD 111,993 (%)	
Age at diagnosis, mean, years ± SD	39.62 ± 14.9	44.4 ± 14.7	<0.001
Age	59.5 ± 15.9	64.95 ± 15.3	<0.001
Age group			<0.001
<50 years	74,233 (74.3)	71,167 (63.5)	
≥50 years	25,729 (25.7)	40,826 (34.7)	
Ethnicity, Arabs	15,154 (15.5)	18,936 (17.3)	<0.001
BMI	29 ± 5.2	30.4 ± 6.6	<0.001
CIHD	27,293 (27.3)	25,007 (22.3)	<0.001
COPD	10,181 (10.2)	11,275 (10.1)	0.372
Asthma	11,302 (11.3)	18,919 (16.9)	<0.001
CRF	14,822 (14.8)	12,511 (11.2)	<0.001
Hypertension	53,255 (53.3)	67,947 (60.7)	<0.001
Diabetes Mellitus	21,562 (21.6)	27,701 (24.7)	<0.001
Dyslipidemia	68,309 (68.3)	79,045 (70.6)	<0.001
Obesity	52,704 (52.7)	71,873 (64.2)	<0.001
CVA	3181 (3.2)	3542 (3.2)	0.798
Dementia	2874 (2.9)	5828 (5.2)	<0.001
Vitamin B12 deficiency anemia	1178 (1.2)	1534 (1.4)	<0.001
Folic acid deficiency	24,208 (24.2)	30,760 (27.5)	<0.001
Iron deficiency anemia	18,267 (18.3)	38,916 (34.7)	<0.001
Cancers			
Lung cancer	1047 (1)	954 (0.9)	<0.001
Prostate	3469 (1.6)	-----	
CRC	1740 (1.7)	1993 (1.8)	0.497
Stomach	350 (0.4)	313 (0.3)	0.004
Breast	68 (0.1)	5481 (4.9)	<0.001
Pancreas	257 (0.3)	294 (0.3)	0.807
Uterus	-----	900 (0.8)	<0.001
Kidney	951 (1)	647 (0.6)	<0.001
Non-Hodgkin lymphoma	736 (0.7)	812 (0.7)	0.762
Hodgkin lymphoma	248 (0.2)	246 (0.2)	0.175
Melanoma	1713 (1.7)	1744 (1.6)	0.005
Basal cell carcinoma	11,546 (11.6)	13,103 (11.7)	0.284
Thyroid carcinoma	368 (0.4)	1175 (1)	<0.001

BMI = Body Mass Index, CIHD = Chronic Ischemic, CVA = Cerebrovascular Accident, COPD = Chronic Obstructive Pulmonary Disease, CRF = Chronic Renal Failure, CRC = Colorectal cancer.

**Table 2:** Laboratory values of females and males included in the study.

Variable	Males 99,962 (47.2)	Females 111,993 (52.8)	*p*-Value
Hemoglobin	14.7 ± 1.38	13.0 ± 1.24	<0.001
WBC	7.8 ± 2.9	7.4 ± 3.1	<0.001
PLT	238 ± 66	266 ± 74	<0.001
AST	33.8 ± 39	30.7 ± 36	<0.001
ALT	47.1 ± 52.9	34.2 ± 38.6	<0.001
GGT	61.45 ± 100	51.89 ± 84	<0.001
Bilirubin	0.5 ± 0.56	0.4 ± 0.4	<0.001
Creatinine	0.95 ± 0.37	0.73 ± 0.29	<0.001
Albumin	4.42 ± 1.76	4.22 ± 1.4	<0.001
Vitamin D	50 ± 23.55	47 ± 25.5	<0.001
Vitamin B12	318 ± 158	352 ± 185	<0.001
Folic Acid	17.6 ± 36.3	19.5 ± 45	<0.001
CRP	3.97 ± 18.6	3.40 ± 16.18	<0.001
Iron	86.6 ± 34	71.5 ± 30	<0.001
Ferritin	177.1 ± 305	89.8 ± 170	<0.001
Calcium	9.47 ± 0.46	9.44 ± 0.48	<0.001
Sodium	140.12 ± 3.2	140.07 ± 3.4	<0.001
INR	1.05 ± 0.34	1.02 ± 0.33	<0.001
APRI	0.44 ± 0.83	0.36 ± 0.66	<0.001
FIB-4	0.96 ± 1.1	1 ± 1.0	<0.001

All values presented as mean ± SD. WBC = White Blood Cells, PLT = Platelets, ALT = Alanine Aminotransferase, AST = Aspartate Aminotransferase, GGT = Gamma-Glutamyl Transferase, INR = International Normalized Ratio, APRI = AST to Platelet Ratio Index.

**Table 3:** Liver-related outcomes and all-cause mortality rates among females and males with NAFLD.

	Males with NAFLD 99,962 (%)	Females with NAFLD 111,993 (%)	
Cirrhosis	1901 (1.9)	2528 (2.3)	<0.001
Age at cirrhosis	63.4 ± 13.7	65.9 ± 12.3	<0.001
HCC	492 (0.5)	451 (0.4)	0.002
Age of HCC	68.39 ± 11	69.33 ± 12	0.227
Esophageal varices	603 (0.6)	675 (0.6)	0.988
Esophageal variceal bleeding	326 (0.3)	321 (0.3)	0.1
SBP	146 (0.1)	157 (0.1)	0.721
Hepatorenal syndrome	136 (0.1)	148 (0.1)	0.806
Liver transplantation	112 (0.11)	81 (0.07)	0.003
Age at liver transplantation	54.96 ± 11.6	55.14 ± 12.4	0.920
Death	10,219 (10.2)	12,744 (11.4)	<0.001
Age at death	74.4 ± 12.8	77.5 ± 11.7	<0.001
Number of hospitalizations From diagnosis, mean ± SD	3.99 ± 6.6	4.65 ± 6.5	<0.001
Length of hospitalization mean ± SD	3.67 ± 11	3.6 ± 9.4	0.154

**Table 4:** Cirrhosis rate according to age group among females and males with NAFLD.

Cirrhosis	Females, n = 111,993			Males, n = 99,962		
Age Group Years	Patient Number (%)	Cirrhosis Number	Cirrhosis Rate per 1000	Patient Number	Cirrhosis Number	Cirrhosis Rate per 1000
18–24	647 (0.6)	1	1.5	716 (0.7)	3	4.2
25–34	4371 (3.9)	13	3	5837 (5.8)	15	2.6
35–44	9271 (8.3)	37	4	15,690 (15.7)	60	3.8
45–54	13,739 (12.3)	112	8.2	18,729 (18.7)	126	6.7
55–64	22,945 (20.5)	336	14.6	18,758 (18.8)	317	16.9
65–74	30,479 (27.2)	755	24.8	21,580 (21.6)	552	25.6
75+	30,541 (27.3)	1274	41.7	18,652 (18.7)	828	44.4
Total	111,993	2528	22.6	99,962	1901	19

**Table 5:** Age-adjusted mortality rates among females and males with NAFLD.

Mortality	Females, n = 111,993			Males, n = 99,962		
Age Group Years	Patient Number (%)	Death Number	Death Rate per 1000	Patient Number (%)	Death Number	Death Rate per 1000
18–24	647 (0.6)	3	4.6	716 (0.7)	2	2.8
25–34	4371 (3.9)	22	5	5837 (5.8)	23	3.9
35–44	9271 (8.3)	48	5.2	15,690 (15.7)	110	7
45–54	13,739 (12.3)	170	12.4	18,729 (18.7)	255	13.6
55–64	22,945 (20.5)	626	27.3	18,758 (18.8)	830	44.2
65–74	30,479 (27.2)	2037	66.8	21,580 (21.6)	2021	93.7
75+	30,541 (27.3)	9839	332.2	18,652 (18.7)	6978	364.1
Total	111,993	12,744	113.8	99,962	10,219	102.2

**Table 6:** Univariate and multivariate analyses of risk factors for cirrhosis among NAFLD patients.

Multivariate Analysis—Males				Multivariate Analysis—Females		
	OR	95% CI	*p*-Value	OR	95% CI	*p*-Value
Age at diagnosis	1.038	1.035–1.042	<0.001	1.032	1.029–1.035	<0.001
Diabetes Mellitus	3.331	3.005–3.692	<0.001	3.403	3.117–3.714	<0.001
Obesity	1.126	1.021–1.241	0.017	0.960	0.880–1.048	0.363
Hypertension	1.032	0.910–1.171	0.620	1.000	0.894–1.120	0.996

**Table 7:** Univariate and multivariate analyses of risk factors for death among NAFLD patients.

Univariate Analysis				Multivariate Analysis		
	OR	95% CI	*p*-Value	OR	95% CI	*p*-Value
Age at diagnosis	1.128	1.126–1.130	<0.001	1.125	1.124–1.127	<0.001
Gender (female)	1.128	1.097–1.159	<0.001	1.382	1.336–1.429	<0.001
Diabetes Mellitus	5.069	4.927–5.215	<0.001	2.648	2.562–2.737	<0.001
Cirrhosis	8.271	7.784–8.788	<0.001	4.016	3.690–4.372	<0.001
Hepatocellular Carcinoma	18.761	16.333–21.550	<0.001	9.086	7.646–10.797	<0.001
Esophageal Varices	10.249	9.172–11.453	<0.001	2.021	1.734–2.357	<0.001

## Data Availability

No additional data are available.
